# Cell Cycle Regulation by Ca^2+^-Activated K^+^ (BK) Channels Modulators in SH-SY5Y Neuroblastoma Cells

**DOI:** 10.3390/ijms19082442

**Published:** 2018-08-18

**Authors:** Fatima Maqoud, Angela Curci, Rosa Scala, Alessandra Pannunzio, Federica Campanella, Mauro Coluccia, Giuseppe Passantino, Nicola Zizzo, Domenico Tricarico

**Affiliations:** 1 Section of Pharmacology, Department of Pharmacy-Pharmaceutical Sciences, University of Bari, Via Orabona 4, 70125 Bari, Italy; fatima.maqoud@uniba.it (F.M.); angela.curci@uniba.it (A.C.); rosa.scala@uniba.it (R.S); 2 Section of Anatomy Pathology, Department of Pharmacy-Pharmaceutical Sciences, University of Bari, Via Orabona 4, 70125 Bari, Italy; Alessandra.pannunzio@uniba.it (A.P.); federica.campanella@uniba.it (F.C.); mauro.coluccia@uniba.it (M.C.); 3 Section of Anatomy Pathology, Department of Veterinary Medicine, University of Bari, Via Orabona 4, 70125 Bari, Italy; giuseppe.passantino@uniba.it (G.P.); nicola.zizzo@uniba.it (N.Z.)

**Keywords:** Ca^2+^-activated K^+^(BK) channel, calcium ions, cell cycle, AKT, cell volume

## Abstract

The effects of Ca^2+^-activated K^+^ (BK) channel modulation by Paxilline (PAX) (10^−7^–10^−4^ M), Iberiotoxin (IbTX) (0.1–1 × 10^−6^ M) and Resveratrol (RESV) (1–2 × 10^−4^ M) on cell cycle and proliferation, AKT1p^Ser473^ phosphorylation, cell diameter, and BK currents were investigated in SH-SY5Y cells using Operetta-high-content-Imaging-System, ELISA-assay, impedentiometric counting method and patch-clamp technique, respectively. IbTX (4 × 10^−7^ M), PAX (5 × 10^−5^ M) and RESV (10^−4^ M) caused a maximal decrease of the outward K^+^ current at +30 mV (Vm) of −38.3 ± 10%, −31.9 ± 9% and −43 ± 8%, respectively, which was not reversible following washout and cell depolarization. After 6h of incubation, the drugs concentration dependently reduced proliferation. A maximal reduction of cell proliferation, respectively of −60 ± 8% for RESV (2 × 10^−4^ M) (IC50 = 1.50 × 10^−4^ M), −65 ± 6% for IbTX (10^−6^ M) (IC50 = 5 × 10^−7^ M), −97 ± 6% for PAX (1 × 10^−4^ M) (IC50 = 1.06 × 10^−5^ M) and AKT1p^ser473^ dephosphorylation was observed. PAX induced a G1/G2 accumulation and contraction of the S-phase, reducing the nuclear area and cell diameter. IbTX induced G1 contraction and G2 accumulation reducing diameter. RESV induced G2 accumulation and S contraction reducing diameter. These drugs share common actions leading to a block of the surface membrane BK channels with cell depolarization and calcium influx, AKT1p^ser473^ dephosphorylation by calcium-dependent phosphatase, accumulation in the G2 phase, and a reduction of diameter and proliferation. In addition, the PAX action against nuclear membrane BK channels potentiates its antiproliferative effects with early apoptosis.

## 1. Introduction

The large conductance voltage and Ca^2+^-activated K^+^ (BK) channel is known to regulate cell excitability, proliferation, and migration in response to a variety of stimulus [1,2,3]. Calcium signaling is involved in the release of neurotransmitters, cell excitability, and muscle contractility. Physiological low intracellular calcium levels induce cell proliferation and survivals, while an abnormal enhancement of intracellular calcium associated with the pathophysiological conditions induces cell death apoptosis. The BK channel is a calcium sensor able to regulate different cell functions including the release of neurotransmitters in the Central Nervous System (CNS), the modulation of the vascular tone, and Excitation-Contraction Coupling (EC) coupling in skeletal muscle fibers being involved in different pathophysiological functions and diseases [4,5,6]. The BK channel is unique among the superfamily of K^+^ channels; indeed, they are indeed tetramers characterized by a pore-forming α subunit containing seven transmembrane segments (instead of the six found in voltage-dependent K^+^ channels) and a large C terminus composed of two K^+^ conductance domains (RCK domains), where the Ca^2+^-binding sites reside. The pore alpha subunit, coded by a single gene (*Slo1*), is activated by depolarizing potentials and by a rise in intracellular Ca^2+^ concentration. BK channels can be associated with accessory β subunits that play a role in the modulation of BK channel gating. Four β subunits have currently been identified (β1, β2, β3, and β4) and despite the fact that they all share the same topology, it has been shown that every β subunit has a specific tissue distribution and that they modify channel kinetics as well as their pharmacological properties and the apparent Ca^2+^ sensitivity of the α subunit in different ways [5]. Splicing isoforms of the *Slo1* gene and accessory gamma subunits are also important in regulating channel function [7].

It was demonstrated that BK channel is a target for a large variety of toxins and modulators; especially, the pore forming alpha subunit represents the binding-site of these compounds whereas the associated beta 1–4 subunits play a critical role in regulating their binding affinity to the pore [5]. Among these toxins, Iberiotoxin (IbTX) is a minor fraction of the crude venom of Buthus tamulus discovered by Galvez et al. in 1990 [8]. It is a relatively impermanent external channel pore blocker of the BK channel, largely used in structural and functional studies [8,9]. Also, IbTX is characterized by an amino acid chain of the same length than Charybdotoxin (ChTX), consisting of 37 residues that possesses 68% of the sequence identity associated with it. Despite their structural similarities, a multitude of functional studies have demonstrated that IbTX binds to the external mouth of the BK channel with higher affinity than ChTX, as indicated by the lower dissociation rate of IbTX compared with ChTX. The binding of these toxins to the BK channel is very sensitive to the electrostatic interactions, involving several basic residues of toxins and negative charges in the outer vestibule of the channel pore [10,11]. Thus, the surface charge distributions and the three-dimensional structures of toxins are important determinants of their recognition and interactions with BK channels [12].

Instead, the tremorgenic mycotoxin paxilline (PAX) is an extremely potent but non-peptide BK channel blocker [13]. It is characterized by a selectivity and specificity for the BK channel so high, comparable with that of IbTX, that different authors reported a very low nM Kd when it is applied from the internal side in an excised patch [13,14]. More recently, it has been reported that the IC50 for PAX may shift from nM values, when channels are closed, to a value of 10 μM, as maximal Po is approached. Then, these findings suggest a mechanism of inhibition in which the allosteric binding of a single molecule may alter the intrinsic L(0), favoring the occupancy of closed states, with an affinity for the closed conformation greater than the affinity for the open one [15].

Both these toxins are reported to inhibit cell migration and proliferation in a variety of cell lines. For instance, chronic exposure of human malignant glioma cells for 72 h with IbTX induces S phase accumulation, reducing cell proliferation [16]. PAX reduces cell proliferation of the human breast cancer MDA-MB-453 following 72 h of incubation time [17] and it is reported to inhibit cell migration in the micromolar concentration range in the malignant pleural mesothelioma [3]. Moreover, in human cardiac c-kit^+^ progenitor cells, this toxin inhibits cell proliferation and leads to accumulation of the cells in G0/G1 phase leading to the inhibition of migration and proliferation following 42–74 h of incubation [18].

Other than IbTX and PAX, the unselective Kv/BK channel blocker tetraethylammonium (TEA) and the potassium channel modulator resveratrol (RESV) showed antiproliferative effects. TEA at 10 mM concentration inhibits cell proliferation, leading to the accumulation of oligodendrocyte progenitor cells in the G1 phase [19]. Instead, RESV is an unselective modulator of BK channels, leading to channel activation in skeletal muscle and neurons but inhibiting the BK channels in human neuroblastoma SH-SY5Y cells [7,20,21,22,23]. Its effects have been extensively investigated in vivo on breast, colorectal, liver, pancreatic, and prostate cancers [24]. Thus, it was shown that RESV affects cell growth, apoptosis, angiogenesis and invasion through a variety of death signaling cascades [25]; for example, it is reported to exert cytotoxic action in neuroblastoma cells, down-regulating the AKT signaling [26].

The C-terminus domain of the pore-forming α subunit of the BK channel contains several protein kinase and phosphatase binding motifs associated with various proteins involved in the regulation of channel gating and signaling pathways. Among these, the attention has been focused on RAC-a serine/threonine-protein kinase 1, also named AKT-1. AKT has a central role in cell survival, since it is implicated in the inhibition of apoptosis by phosphorylating and inactivating targets such as BAD and caspase-9 [27]. Recent data suggest a direct physical interaction between AKT and BK channel via AKT binding sites that overlap at the C terminus [28]. In addition, knockdown of AKT-1 increases BK expression, suggesting that BK is kept in check by these kinases. These results also suggest that both these proteins belong to a complex interacting network that regulates cell death or survival [28].

To date, the relationship between BK channel blocking mechanisms and the biological effects associated with cell survivals are poorly described, particularly after a short-term incubation time. In our experiments, we used the high affinity BK channel blockers IbTX and PAX to investigate on the relationship between their blocking mechanisms and biological effects, such as cell cycle progression and the associated intracellular signaling, morphological changes, and cell proliferation. The acute effects of these compounds were investigated after a short incubation time of 6 h, which was the minimal time required to observe the antiproliferative effects of these compounds in SH-SY5Y cells in our experiments. Finally, the effects of RESV and TEA on cell cycle progression and proliferation were also investigated. The effects of these BK channel modulators were investigated in the SH-SY5Y neuroblastoma cells because they express a large BK channel current representing 50% of the outward K^+^ currents. The BK channel also regulates cell proliferation in this cell line [29,30]. The role of the BK channel in this cell is supported by the fact that the downregulation of the gene expression by gene-specific mRNA silencing using RNA interference (RNAi) significantly reduced *Slo1* mRNA expression and cell proliferation after three days of the transfection when compared to non-transfected cells [23].

We found that the early response of BK channels to the external channel pore blocker IbTX induces a contraction of the G1 phase and an accumulation in G2; whereas the allosteric blocker PAX induces the S Phase contraction and accumulation in G1/G2 phase. The two different blockers of the BK channels IbTX and PAX as well as the BK channel modulator RESV share a common action, leading to accumulation in G2 phase, AKT1p^ser473^ dephosphorylation, and a reduction of cell diameter. This can be related with the block of surface membrane BK channels. In addition, the action of PAX against intracellular BK channels and/or the interaction with the allosteric side of the pore can be associated with G1 accumulation, nuclear area reduction, and the potentiation of its antiproliferative effects with early apoptosis.

## 2. Results

### 2.1. Effects of the Application of Kv/BK Channels Targeting Compounds on the Whole-Cell K^+^ Current of SH-SY5Y Cells

**Figure 1:**
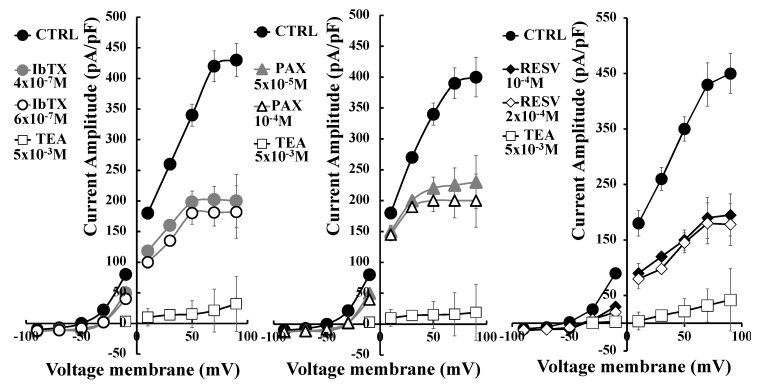
Current-voltage relationship of the whole-cell K^+^ current recorded in SH-SY5Y cells: characterization of the maximal inhibitory effect of the application of the Ca^2+^-activated K^+^ (BK) channel blockers paxilline (PAX) and iberiotoxin (IbTX). A sigmoid I/V relationship of the K^+^ current in a single SH-SY5Y cell recorded in the control condition (CTRL) and in the presence of the specific BK channel blockers PAX at 5 × 10^−5^ and 1 × 10^−4^ M concentrations, IbTX at 4 × 10^−7^ and 6 × 10^−7^ M and RESV at 10^−4^ and 2 × 10^−4^ M concentrations. The experiments were performed in asymmetrical K^+^ ion concentrations (int K^+^: 1.32 × 10^−1^ M; ext K^+^: 2.8 × 10^−3^ M) and in the presence of an internal concentration of free Ca^2+^ ions of 1.6 × 10^−6^ M. Membrane currents were activated by 500 ms voltage steps between −90 to +90 mV, starting from an HP of −60 mV (Vm). The leak was obtained through the application of saturating concentration of the K^+^ channel blocker tetraethylammonium (TEA) (5 × 10^−3^ M). Cells characterized by the same size were selected for patch clamp experiments. Each point represented the mean ± SEM of 5–8 different experimental points.

As previously shown, the SH-SY5Y cells showed an outward current carried by K^+^ ions [30]. The I/V relationship intercepted the voltage axes at −49 ± 4 mV (Vm) (Figure 1) in the control condition. This value represented the resting membrane potential characterizing these cells, according to the well-described experimental observation that they show a more depolarized resting membrane potential than their normal counterpart, because of their cancerous nature [31]. These values represented the resting membrane potential characterizing these cells, according to the well-described experimental observation that they show a more depolarized resting membrane potential than their normal counterpart, because of their cancerous nature [31]. IbTX at 4 × 10^−7^ M concentration (*N* of cells = 7) and PAX at 5 × 10^−5^ M concentration (*N* of cells = 8) caused a comparable decrease of the whole-cell K^+^ current at +30 mV (Vm) of −38.3 ± 10% for IbTX and −31.9 ± 9% for PAX, respectively, and were significantly different vs controls as evaluated by the Student’s *t*-test (n.s. PAX data vs IbTX data; *p* < 0.05 PAX and IbTX vs controls). No further decrease of the current was observed at a higher toxin concentration. The maximal inhibitory response was −38.6 ± 8% for PAX at 1 × 10^−4^ M concentration (*N* of cells = 5) and −45.8 ± 8% for IbTX at 6 × 10^−7^ M concentration (*N* of cells = 5) significantly different vs controls as evaluated by the Student’s *t*-test (n.s. PAX data vs IbTX data; *p* < 0.05 PAX and IbTX vs controls). The unselective BK modulator RESV also induced a marked reduction of the whole-cell K^+^ current of −63.7 ± 15% (*N* of cells = 5) at 2 × 10^−6^ M concentration in this cell line. The IbTX (6 × 10^−7^ M), PAX (1 × 10^−4^ M), and RESV (2 × 10^−4^ M) also shifted the I/V relationships to the right on the voltage axis; for instance, the resting membrane potential was −29 ± 4, −31 ± 5, and −27 ± 3 mV for IbTX, PAX and RESV, respectively (Figure 1). The addition of the broad-spectrum K^+^ channel blocker TEA at the saturating concentration of 10^−3^ M caused in all experiments a full reduction of the total K^+^ current of the −93 ± 11%, allowing the definition of the leak current. At +100 mV (Vm) the current reduction by both toxins was more marked than that calculated at +30 mV (Vm), supporting the idea that the BK channel current component represented about 35–50% of the total whole-cell K^+^ current in this cell line.

**Figure 2:**
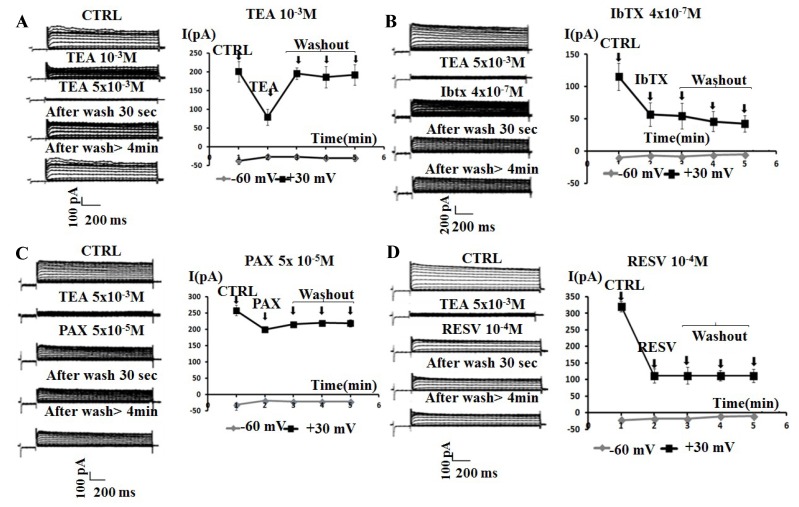
Sample traces of the whole-cell K^+^-current and time courses recorded in SH-SY5Y cells in the CTRL condition (CTRL) and following the addition of the Kv/BK channel blockers TEA, IbTX, PAX, and resveratrol (RESV) to the bath solution. The current was recorded in asymmetrical K^+^ ions concentrations (int K^+^: 1.32 × 10^−1^ M; ext K^+^: 2.8 × 10^−3^ M) and in the presence of an internal concentration of free Ca^2+^ ions of 1.6 × 10^−6^ M. Membrane currents were activated by 500 ms voltage steps between −100 to +100 mV, starting from an HP of −60 mV (Vm). The leak current was recorded in the presence of a saturating concentration of TEA (5 × 10^−3^ M). The four panels describe the effects of the application of the Kv/BK channel blockers under investigation on cells, according to the following scheme: current traces recorded in the control condition (CTRL); current traces recorded in the presence of TEA (5 × 10^−3^ M); current traces recorded after the application of specific concentration of Kv/BK channel targeting compounds under investigation: (**A**) TEA 5 × 10^−3^ M; (**B**) IbTX 4 × 10^−7^ M; (**C**) PAX 5 × 10^−5^ M; (**D**) RESV 10^−4^ M); current traces recorded following two washout periods with the bath solution of 30 s and >4 min. Time courses of the K^+^ current in the presence or absence of the compounds under investigation recorded at physiological Vm of +30 and −60 mV. Each point is the mean + SEM of 3 cells. The incubation of the cells with TEA induced almost a total reduction of the current, which was fully reversible within 30 s of washout with bath solution. In contrast, the incubation of cells with IbTX, RESV, and PAX induced a partial reduction of the total K^+^ current, which was not reversible within 4 min of washout with bath solution.

The inhibitory effect of TEA was fully reversible within 30 s of washout of the bath solution (Figure 2A). While, the effects of the application of the BK blockers IbTX and PAX on the whole-cell K^+^-current were not reversible within 30 s of washout of the bath solution (Figure 2B,C). A washout period with bath solution >4 min failed to restore the K^+^-currents to the control values. Similarly, the inhibitory effect of the not selective BK modulator RESV was not reversible following washout (Figure 2D, Table 1).

In addition, we tested the possible effects of the patch perfusion with the bath solution on the recorded K^+^ currents, reducing or increasing it by influencing the seal stability. In the control condition (*N* of cells = 15), the K^+^-currents were stable all over the observation period (8 min).

The application of DMSO (8.4 × 10^−10^%–1.7 × 10^−1^%) to SH-SY5Y cells tested at the percent concentrations (*v*/*v*) corresponding to the amount used as a co-solvent caused a not significant decrease of K^+^-currents with respect to that of the controls recorded at +30 mV (Vm) (*I* mean recorded after DMSO washout vs *I* mean recorded in control condition, Student’s *t*-test *p* > 0.05).

### 2.2. Cell Proliferation and Cell Cycle Progression in SH-SY5Y Cell Following 6 h Incubation of the Cells with Kv/BK Channel Targeting Drugs

**Figure 3:**
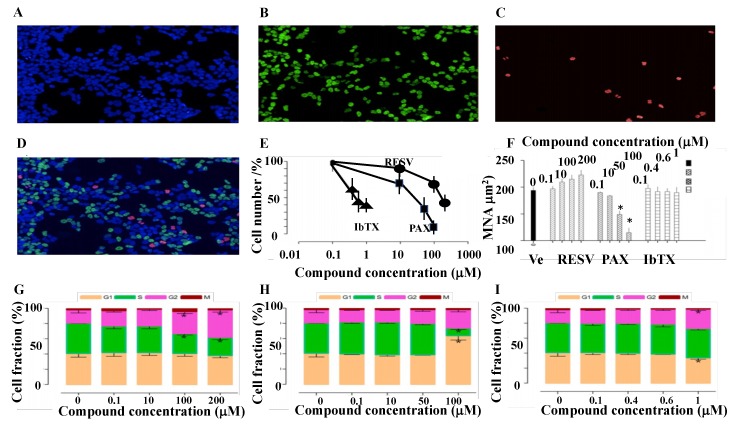
High-content cell-cycle analysis. (**A**–**D**) Example images of vehicle (DMSO 1.7 × 10^−1^%)-treated SH-SY5Y cells stained with (**A**) NuclearMask Blue Stain, and (**B**) Alexa Fluor 488 (green, S-phase cells), or (**C**) anti-pHH3 (red, M-phase cells) antibodies are shown. (**D**) Merged image from panels (**A**–**C**). The effects of RESV, PAX, and IbTX treatment upon cell number and nuclear area are shown in panels (**E)** and (**F**), respectively. Concentration-response cell-cycle modifications by RESV, PAX, and IbTX determined using the multi-parametric approach are shown in panels (**G**–**I)**. Values are the mean ± SD of 5 biological replicates; * *p* < 0.05 versus vehicle-treated controls (Student’s *t*-test, two-tailed distribution, two sample equal variance). Operetta high-content imaging system (Perkin Elmer Life Sciences, Boston, MA, USA) was performed using a 20× long working distance objective. Typically, 10 fields per well were imaged which equated to approximately 6000 cells/well.

Cell proliferation evaluated using the impedentiometric technique (Scepter cell counter) revealed no effects of the compounds under investigation within 1–4 h of incubation time. These compounds failed to affect the cell cycle after 4 h of incubation. After 6 h of incubation the cell proliferation-time relationship for this cell line linearly increased with time. We therefore investigated the effects of these compounds after 6 h of incubation on cell cycle progression and proliferation. The cell-cycle changes induced by PAX, IbTX, and RESV tested in the range of concentrations inhibiting K^+^ currents on SH-SY5Y cells have been investigated by the Operetta high-content imaging system, using a multiparametric approach to quantify DNA content and nuclear morphological properties (NuclearMask™ Blue Stain), DNA synthesis (EdU-positive cells), and mitotic cells (pHH3-positive cells). The vehicle DMSO (8.4 × 10^−10^%–1.7 × 10^−1^%) did not significantly affect the investigated parameters (Figure 3A–D). The effect of PAX, IbTX, and RESV on cell cycle and proliferation was investigated and compared by identifying, G1, S, G2, and M cell populations (Figure 3E–I). PAX, IbTX, and RESV reduced cell number in a concentration-dependent way and are characterized by a different inhibitory potency (Figure 3E); assuming that the application of increasing drug concentrations would be capable to induce a total reduction of cell number, the calculated IC_50_ values were 1.06 × 10^−5^, 5 × 10^−7^ and 1.5 × 10^−4^ M, respectively. IbTX (4 × 10^−7^ M) (*N* of experiments = 5) and PAX (5 × 10^−5^ M) (*N* of experiments = 5) respectively caused −40 ± 8% and −65 ± 4% significant reduction of cell proliferation vs vehicle (Ve) treated cells as evaluated by Student’s *t*-test (PAX vs IbTX *p* < 0.05; PAX and IbTX vs vehicle, *p* < 0.05). A maximal reduction of cell proliferation respectively of −62 ± 7% and −65 ± 6% and for IbTX at 6 × 10^−7^ and 10^−6^ M concentrations, and of −97 ± 6% for PAX (1 × 10^−4^ M); PAX data appeared to be statistically different vs IbTX data as determined by Student’s *t*-test (*p* < 0.05). RESV at 2 × 10^−4^M caused a reduction of cell proliferation of −60 ± 8% (*N* of cells = 5). The effect on cell number was accompanied by modifications of nuclear morphological features in the case of RESV and PAX, whereas IbTX did not produce significant modifications. RESV and PAX produced opposite effects, showing a concentration-dependent nuclear area increase and decrease, respectively. The nuclear shrinkage induced by PAX at 10^−4^ M concentration was associated to an increase of nuclear fragmentation index, calculated from the coefficient of variance (CV) of nuclear stain intensity, indicative of apoptosis induction. All compounds induced cell cycle progression alterations, with a different pattern. In detail, the unselective permeable BK channel modulator RESV induced a G2 phase accumulation accompanied by a contraction of S-phase cells at 10^−4^ and 2 × 10^−4^ M. The selective membrane permeable BK channel blocker PAX also induced G2 accumulation and S contraction, and additionally, a G1-phase accumulation at 10^−4^ M. Finally, the selective external membrane impermanent BK channel blocker IbTX at 10^−6^ M concentration induced a G2-phase accumulation along with a slight contraction of G1 cells. Notably, IbTX is the only one for whom the fraction of cells actively synthesizing DNA was not reduced by treatment, thus suggesting a distinct mechanism of action (Figure 3G–I, Table 1).

### 2.3. Cell Volume Changes Induced by Kv/BK Channels Targeting Compounds

**Figure 4:**
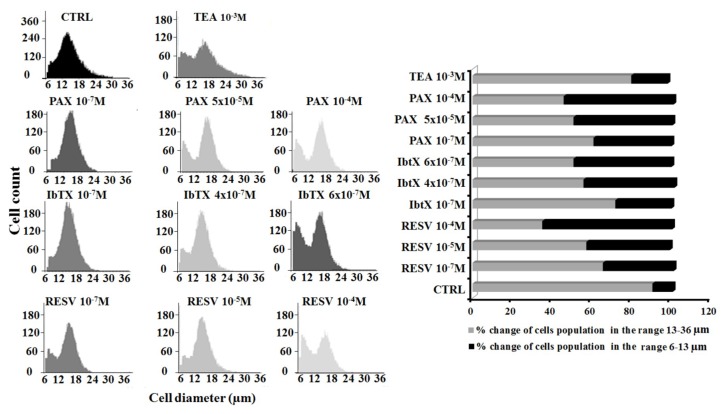
Gaussian type distribution of SH-SY5Y cells as a function of cell diameter size in the presence or absence of Kv/BK channel blockers: effects of the drugs on cell diameter after 6 h of incubation time. The effects of PAX (10^−7^, 5 × 10^−5^ and 10^−4^ M), IbTX (10^−7^, 4 × 10^−7^ and 6 × 10^−7^ M), RESV (10^−7^, 10^−5^ and 10^−4^ M) and TEA (10^−3^ M) on cell distribution according to their diameter size were investigated in SH-SY5Y cells. The SH-SY5Y cell distribution follows a Gaussian curve when plotted as a function of their diameter size. Each graph is representative of cell distribution in a specific experimental condition. A biphasic distribution was observed after 6 h incubation time of cells with PAX, IbTX, and RESV. All compounds induced a concentration-dependent reduction of the cell numbers in the physiological diameter range of 13–36 μm for this cell type vs controls excepts TEA, while increasing the proportion of cell population in the diameter range of 6–13 μm.

To test the hypothesis that the observed effects on cell proliferation and cell cycle progression observed after 6 h of incubation may be associated to cell volume changes, the effects of the Kv/BK channel blockers on cell volume were evaluated using an impedentiometric method (Scepter cell counter). The distribution of SH-SY5Y cells as a function of their diameter size was well described by a Gaussian curve. In the CTRL condition, the majority of cells showed a diameter size in the range between 13–18 μm, which can be considered, for this cell type, the diameter range of normal cell dimensions (Figure 4). The application of the Kv/BK channels targeted compounds under investigation altered this cell distribution (Figure 4). In particular, the application of increasing concentrations of PAX (10^−7^–10^−4^ M) reduced the number of cells in the range between 13–36 μm and determined the appearance of an additional peak in the range of diameter size between 6 and 12 μm. PAX induced a concentration-dependent increase of the cell population showing a diameter size between 6 and 12 μm vs controls. The same effects were observed following the incubation of SH-SY5Y cells with increasing a concentration of IbTX (10^−7^–6 × 10^−7^ M) and RESV (10^−7^–10^−4^ M).

We compared the effects of the BK channel targeting compounds under investigation with that of the unspecific K^+^ channel blocker TEA to underline differences in the behavior of cells exposed to a BK selective *vs* a broad spectrum Kv/BK channels blocker TEA. The application of the TEA (10^−3^ M) did not reduce the total number of cells and did not significantly affect their diameter size vs controls (Table 1).

### 2.4. AKT1p^ser473^a Putative Node in the Signaling Pathways Regulating Cell Proliferation in Response to the Modulation of the BK Channel Activity

**Figure 5:**
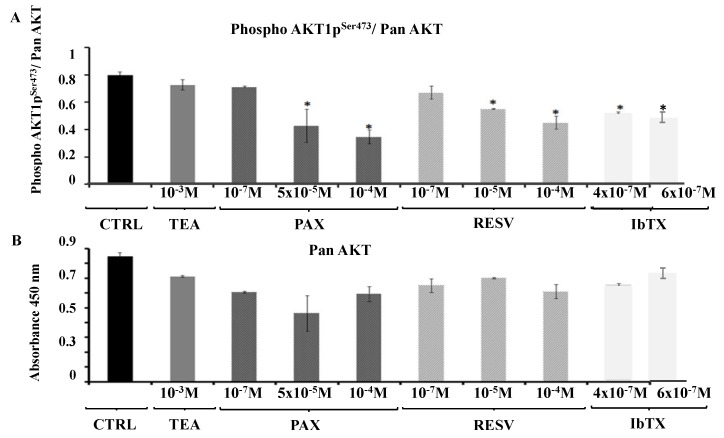
Effects of the Kv/BK channel blockers on the total AKT expression (Pan AKT) and PhosphoAKT (Ser473)/ Pan AKT ratio in SH-SY5Y neuroblastoma cells. The PhosphoAKT (Ser473) and the Pan AKT levels were evaluated in the cell lysates using the ELISA kit for PhosphoAKT (Ser473)-AKT pan (SIGMA Chemical Co., Milan, Italy) in the CTRL condition and after 6 h of incubation with PAX (10^−7^, 5 × 10^−5^ and 10^−4^ M), IbTX (4 × 10^−7^ M), RESV (10^−7^, 10^−5^ and 10^−4^ M) and TEA (10^−3^ M). (**A**) The incubation of cells with PAX, IbTX, and RESV significantly reduced the PhosphoAKT (Ser473) levels. (**B**) No effects were observed on the pan AKT levels reducing the PhosphoAKT (Ser473)/pan AKT ratio. The represented bars correspond to the mean of three different experimental data ± SEM. * Data significantly different with respect to the CTRL condition for *p* < 0.05 as determined by one-way ANOVA and Bonferroni test.

Finally, we tested the hypothesis that the downstream signaling pathway activated by the BK channel targeting compounds under study and resulting in the observed modulation of SH-SY5Y cell viability, may involve the serine-threonine kinase AKT-1. The incubation of the SH-SY5Y cells over a 6 h period with the Kv/BK channels targeting compounds under investigation affect the PhosphoAKT (Ser473) (AKT1p^ser473^)/ Pan AKT ratio. In particular, the incubation of cells with RESV (10^−7^, 10^−5^ and 10^−4^ M) caused a reduction of the PhosphoAKT (Ser473)/ Pan AKT ratio at the higher tested concentration. Also IbTX (4 × 10^−7^, 6 × 10^−7^ M) and PAX (10^−7^, 5 × 10^−5^ and 10^−4^ M) at the higher tested concentrations caused a significant reduction of PhosphoAKT (Ser473)/Pan AKT ratio; statistical analysis performed by One way ANOVA (*p* value = 0.0215, *F* value = 6.37) and Bonferroni test to assess the experimental groups statistically different from the CTRL condition, (*p* < 0.05) (Figure 5A).

The Pan AKT values were not significantly affected by PAX, IbTX, and RESV, indicating that the observed reduction of the PhosphoAKT (Ser473)/Pan AKT ratio was due to the reduction of the PhosphoAKT (Ser473) value (Figure 5B). In contrast, the incubation with TEA (10^−3^ M), the only compound that was capable to induce a reversible decrease of the K^+^ current, failed to affect both Pan-AKT and the PhosphoAKT (Ser473)/ Pan AKT ratio (Figure 5A,B, Table 1).

IbTX (4 × 10^−7^ M) and PAX (1 × 10^−5^ M) at concentrations that fully blocked membrane surface BK channels induced AKT1p^Ser473^ dephosphorylation of 33 ± 5% and 50 ± 8% vs controls, respectively. A mild potentiation of the dephosphorylation is observed at higher toxins concentrations.

## 3. Discussion

In our experiments we used the high affinity BK channel blockers PAX and IbTX to investigate on the relationship between their blocking mechanisms and biological effects such as cell cycle progression and the associated intracellular signaling, the morphological changes, and cell proliferation after a short incubation time. The incubation time of 6 h was the minimal time required to observe the antiproliferative effects of these compounds in this cell line. No effects were indeed observed following 1–4 h of incubation of the cells with IbTX, PAX, and RESV.

IbTX is considered a not permeable BK channel blocker interacting on the external binding site of the pore and acting as an open channel blocker; tested at 4 × 10^−7^ and 6 × 10^−7^ M concentrations it caused a partial whole-cell K^+^ current reduction and a full block of the membrane BK channels, leading to a partial reduction of cell proliferation and AKT1p^ser473^ dephosphorylation. No further effects were observed with IbTX at the highest concentration tested of 10^−6^ M.

PAX is instead considered a membrane permeable and allosteric blocker of BK channels; tested at concentrations of 5.0 × 10^−4^ and 10^−4^ M it caused a partial reduction of the whole-cell K^+^ current and a full block of membrane BK channels, leading to a fully reduced cell proliferation and to a marked AKT1p^ser473^ dephosphorylation. These findings suggest that additional targets are involved in the PAX action other than the membrane BK channels.

The differences in the IC50 values of IbTX and PAX in inducing antiproliferative effects can be explained by the different binding affinity of the toxins to the BK channels site. Indeed, the placement of the binding site of these compounds plays a key role, since it is located on the intracellular side of the channel for PAX and on the extracellular portion of the pore for IbTX [9,15,32]. In this way, IbTX interaction with its binding site is expected to be faster than that of PAX, considering that the access to the binding site of PAX can be limited by the transmembrane flux, and consequently explaining why smaller concentrations of IbTX are required for exerting its own effects on cell proliferation.

Further insights into the toxins interaction with the BK channels may include the molecular composition of the channel. In fact, the β4 subunit leads to a BK channel phenotype that is characterized by a very low sensitivity to ChTX and IbTX [5]. For instance, the presence of the auxiliary β_4_ subunit dramatically decreases the BK channel sensitivity to IbTX by inducing a marked rise in the apparent half-maximal inhibitory concentration (IC_50_) of the BK channel and a decrease of the IbTX association rate to it [33]. The PAX-BK channel interaction observed in our experiments could probably be affected by the presence of the auxiliary β4 subunit, which could modify channel properties, such as its kon and koff kinetics and the pharmacological features.

Actually, the β4 subunit seemed to be involved also in the modification of the toxins’ dissociation from the BK channel complex [5]. In fact, it has been demonstrated that IbTX block of the BK channel takes place through a reversible bimolecular mechanism by which it can bind the external portion of the channel [9,32]. Moreover, also the mechanism by which PAX binds to the BK channel has been well characterized as inversely related to the open probability (Po) of the BK channel and completely reversible when applied from the internal side in excised-patch experiments [15]. On the contrary, in our experiments, the binding of both toxins as well as of RESV to the BK channel is irreversible. This effect can be likely explained by the presence of the modulatory β4 subunit in the SH-SY5Y cells as previously proposed [5].

Instead, the mechanism of interaction of RESV against the BK channel is not known. This natural compound was found to increase the BK channel activity in human vascular endothelial cells, when tested in whole cell patch, and in rat skeletal muscle fibers, in excised patch, thereby proposing this compound in neuromuscular disorders as a BK channel opener [6,7,34,35,36,37]. Others found a significant reduction of both the inward and the outward K^+^ currents in pancreatic β cells by RESV [38,39,40].

Toxins and RESV induced also a concentration dependent reduction of the number of the cell population in the mean diameter range of 13–36 μm, which is the physiological range for healthy cells, accompanied by an increased number of suffering cells showing a mean diameter range of 6–12 μm. Either the selective BK channel blockers IbTX and PAX induced early an accumulation in the G2 phase and AKT1p^ser473^ dephosphorylation after 6 h of incubation time with a reduction of cell proliferation. Similarly, RESV, which is a membrane permeant but not selective BK channel modulator, induced G2 accumulation and AKT1p^ser473^ dephosphorylation reducing cell proliferation. These findings suggest that G2 accumulation is associated with AKT1p^ser473^ dephosphorylation following the acute but not reversible membrane channel block.

PAX and RESV also induced the contraction of the S phase, which is not observed with IbTX in our experimental condition. A possible interaction with intracellular BK channels located on the mitochondrial membrane and nuclear membrane can be associated with a reduced synthetic S phase. An additional G1 accumulation is observed with PAX, which is also capable to induce nuclear fragmentation and a reduction of the nuclear area, a well-known index of apoptosis. This effect can be associated with the interaction of PAX with the intracellular binding site located on the BK channel. In the brain for instance the BK channel interacts with proteins placed in cellular compartments other than in the plasma membrane. Recent studies revealed that the majority of BK channel interactome belongs to proteins localized, in a decreasing order, respectively in the cytoplasm, plasma membrane, nucleus, mitochondria, extracellular region, Golgi apparatus and endoplasmic reticulum [41]. PAX was therefore the most effective compound in our experiments showing G1/G2 accumulation and leading to −95% reduction of cell proliferation. The accumulation in G1 at the end of the first checkpoint is critical in making the decision if a cell should enter the S phase and divide, delay division, or enter G0. The accumulation in G2 at the second checkpoint limits the number of cells to progress into the mitosis. Several compounds acting as antiproliferative agents accumulate cells in G1 and G2 phases; for example 5-flurouracile and rapamicine induces accumulation in G1 phase whereas paclitaxel and other antimitotic agents accumulate in the G2/M phase [42].

G1/S transition is associated with upregulation of several types of K^+^ channels that leads to the hyperpolarization of the cell membrane whereas intracellular blocking action may lead to G1 accumulation, as observed in our experiments with PAX [43]. The accumulation in G2 induced in our experiments by potassium channel blocking drugs like IbTX, PAX, and RESV is expected since the transition from G2 to M is associated with depolarization. During the M phase an elevated K^+^ conductance is reported in several cell types [31]. Consequently, this is in line with the proposed role of the BK channel on the cell cycle. The BK channel can regulate cell volume, provide a driving force for Ca^2+^ entry, hyperpolarize the cell at the G1/S transition, depolarize it towards G2/M one and finally sustain the K^+^ conductance during the M phase [31]. Calcium ions are indeed a key regulator of cell cycle progression and are required in the S phase, leading to cell proliferation at physiological concentrations. Abnormal enhancement of intracellular calcium may however induce calcium dependent apoptosis. Additionally, non-canonical, permeation-independent mechanisms may be involved, where BK channels recruit or modulate signaling cascades via protein–protein interactions. It is tempting to assume that signaling cascades activated by such interactions could link the nuclear clock control with its cytoplasmic counterpart [43].

Potassium channels regulate cell volume along the cell cycle [44]. Indeed, the elevated intracellular K^+^ concentration increases the turgor pressure and cell diameter, which is required for cell growth. So, this is achieved by the activity of the inward rectifier K^+^ channels. Instead, mitosis is associated to a decrease in turgor pressure owing to the K^+^ efflux through the Kv/BK channels [43]. An increase in cell volume is expected very early following the drug blocking action of potassium channels that precede apoptosis [45]. In our experiments, toxins and RESV reduced the cell volume after 6 h of incubation and a lower intracellular K^+^ ion concentration is expected when the apoptotic signaling are ongoing.

We focused on the capability of the drugs under investigation to alter the activity of AKT-1, a serine/threonine kinase that has emerged as a critical signaling node in the pathways that regulate principal cellular functions and activities such as cell survival, growth, proliferation, angiogenesis, metabolism, and migration in several types of cells [27]. This Ca^2+^ regulated kinase belongs to the PI3K/AKT pathway involved in the neuroblastoma development [46,47] for both primary neuroblastoma cells and cell lines, included also SH-SY5Y cells. This signaling pathway promotes cell survival and proliferation by activating survival associated proteins and by inhibiting the apoptotic pathway [48,49,50]. Moreover, a recently published BK channel sequence scanning, aiming to point out conserved BK channel interactions involving apoptosis, Ca^2+^ binding, and trafficking, in chicks, mice, and humans [28], revealed that a potential AKT-1 substrate binding site is present on the BK channel sequence and is highly conserved among species. AKT-1 mediated phosphorylation of the BK channel alpha subunit leads to a downregulation of the membrane of the BK channel [28]. We investigated if the observed effects induced by the BK channel targeting drugs under study on cell cycle and proliferation may be related with a modulation of AKT-1 activity. Interestingly, PAX, IbTX and RESV but not TEA induced a significant reduction of PhosphoAKT (Ser473), which represented the active form of this kinase [51]. The AKT-1 activation can stimulate cell proliferation through multiple downstream targets impinging on cell-cycle regulation, including the cyclin-dependent kinase inhibitors p27 and p21 that once phosphorylated and are sequestrated in the cytosolic compartment, which avoids their nuclear localization and the possibility of exerting their inhibitory effects on the cell cycle [52,53,54,55]. Other AKT target proteins are represented by GSK3, TSC2, and PRAS40, which are involved in cell-cycle entry and progression [27].

We therefore propose the following cascade of events to explain the antiproliferative effects of the BK channel openers (Graphical abstract). The irreversible blocking action of the surface membrane BK channels by PAX, IbTX, and RESV induce persistent cell depolarization and influx of cations including Ca^2+^ ions. Cell depolarization is observed after acute exposure of the cells to the BK channel modulators in the presence of a micromolar concentration of internal calcium ions. In the presence of lower Ca^2+^ ions concentrations the contribution of the BK channel to the total currents and the effects of the channel blockers on resting potentials is expected to be proportionally reduced. Cell depolarization and cation influx was recently observed in glioma cells following exposure of the cells to Penitrem A, a BK channel blocker [56]. After 6 h of incubation, we believe that the cell depolarization would activate the death calcium dependent signaling with early apoptosis and a reduction of the cell diameter in the case of IbTX, RESV, and PAX. The increase of the intracellular Ca^2+^ ions activate phosphatase such as pleckstrin homology domain leucine-rich repeat protein phosphatase PHLPP that specifically dephosphorylate AKT-1 on Ser473 and with less extended protein phosphatases PP2A that may also dephosphorylate AKT-1 at Ser473 [57]. The cytosolic AKT-1 dephosphorylation is per se responsible for G2 phases accumulation [58]. The nuclear AKT-1 dephosphorylation can be responsible for G1 phases accumulation induced by PAX following interaction with the nuclear membrane BK channel and a reduction of the nuclear membrane area as observed in our experiments.

## 4. Materials and Methods

### 4.1. Drugs

The drugs and toxins were purchased from Sigma (SIGMA Chemical Co., Milan, Italy). Stock solutions of the drugs under investigation were prepared dissolving the drugs in dimethylsulfoxide (DMSO) at the concentration of 1.186 × 10^−1^ M (PAX and RESV) and 1 × 10^−1^ M (IbTX). Microliter amounts of the stock solutions were then added to the bath solutions, for patch clamp experiments, or to DMEM+, for cell proliferation, cell volume and AKT ELISA assays, as needed. DMSO did not exceed 0.017% in the bath or DMEM+; at this concentration the solvent does not normally affect the parameters under study.

### 4.2. Patch Clamp Solutions

Pipette (intracellular) solution composition (10^−3^ M): 132 K^+^-glutamate, 1 ethylenegly-col bis (β-aminoethylether)-*N*,*N*,*N*,*N*-tetraaceticacid (EGTA), 10 NaCl, 2 MgCl_2_, 10 HEPES, 1 Na_2_ATP, 0.3 Na_2_GTP, pH = 7.2 with KOH. CaCl_2_ was added to the pipette solutions to give free Ca^2+^ ion concentration of 1.6 × 10^−6^ M in whole cell experiments. The calculation of the free Ca^2+^ ion concentration in the pipette was performed using the Maxchelator software (Stanford University, Stanford, CA, USA).

Bath (extracellular) solution composition (10^−3^ M): 142 NaCl, 2.8 KCl, 1 CaCl_2_, 1 MgCl_2_, 11 glucose, 10 HEPES, pH = 7.4 with NaOH.

### 4.3. Whole-Cell K^+^ Current Recordings in the SH-SY5Y Cells

The native K^+^-currents were recorded in the human neuroblastoma cell line SH-SY5Y through the application of the voltage protocol by 10 mV and 500 ms voltage steps, in the range of potentials going from −100 mV (Vm) to +100 (Vm) and starting from HP = −60 mV. The experiments were performed in asymmetrical K^+^ ion concentrations (int K^+^: 1.32 × 10^−1^ M; ext K^+^: 2.8 × 10^−3^ M), in the presence of free Ca^2+^ ions, using a patch-clamp technique in the whole cell configuration [59,60]. The resulting K^+^-currents were leak subtracted. The leak current was measured after adding the saturating concentration of an external solution containing TEA (1 or 5 × 10^−3^ M), which caused a full block of the Kv and BK channels. Drug effects were investigated in a physiological range of potentials going from −80 mV (Vm) to +30 mV (Vm) for all drugs. The K^+^-currents were recorded at 20 °C and sampled at 2 kHz (filter = 1 kHz) using an Axopatch-1D amplifier equipped with a CV-4 headstage (Axon Instruments, Foster City, CA, USA) as previously described [4,61,62].

Current analysis was performed using pCLAMP 10 software package (Axon Instruments, Foster City, CA, USA). The criteria for accepting the data entering were based on the stability of the seal evaluated by observing the noise levels not exceeding 0.6 pA. The pipettes resistance was 7 ± 0.2 MΩ. The cells were exposed to the drug solutions for 1 min. before recordings. In patch clamp experiments, the bath solution was tested against K^+^-currents in the absence of the control, in the presence of DMSO, and in the presence of the DMSO + drug. Increasing concentrations of drug solutions were applied to the cells by the fast perfusion system (AutoMate, Sci. Berkeley, CA, USA). No more than six different solutions were applied to the same cell. Seal resistance was continuously monitored during the patch solutions exchange. The reversibility of the drug response of the currents was evaluated in the presence or absence of compounds plotting raw data vs time in cells of different size. This type of experiment required the continuous monitoring of seal resistance for 5 min before the application of the compound. Cells not showing a stable seal during monitoring were disregarded and were not selected for the further analysis. The data reported in Figure 2 were pooled from three cells for each compound that reached a stable seal for at least 5 min before the application of the compound. By using this protocol we had a very low S.E.M. for each time point.

### 4.4. Cell Culture

The human neuroblastoma cell line SH-SY5Y (ATCC^®^ CRL2266™) were purchased from American Type Culture Collection (ATCC, Manasass, VA, USA). The cells were cultured in Dulbecco’s Modified Eagle’s Medium (DMEM) supplemented with 10% fetal bovine serum (FBS), 2 mM L-glutamine and 1% (100 IU/mL penicillin and 100 μg/mL streptomycin) antibiotics. The cultures were maintained at 37 °C in a humidified atmosphere containing 95% air and 5% CO_2_ in a humidified atmosphere containing 5% CO_2_. All culture medium components were purchased from EuroClone (EuroClone S.p.A, Pero, Italy). The experiments were performed on undifferentiated cells at a passage comprised between 10 and 14 during the experiments.

### 4.5. High-Content Cell Cycle Analysis

Treatment-induced modifications of cell cycle phases were investigated using the high-content imaging system Operetta (Perkin Elmer Life Sciences, Boston, MA, USA), along with Harmony software and PhenoLOGIC machine learning, according to a method recently described by Massey [63]. Briefly, SH-SY5Y human neuroblastoma cells at passage 10 were seeded (6500 cells/well) in DMEM (Dulbecco’s modified Eagle’s medium) complete culture medium with 10% fetal bovine serum, *L*-glutamine and Penicillin/Streptomicin (cell biology reagents from EuroClone S.p.A, Pero, Italy) in 96-well collagen-coated plates (Perkin Elmer). Cells were allowed to attach for 24 h in a normal humidified atmosphere supplemented with 5% CO_2_, and then treated in the same conditions for 6 h with different concentrations of freshly dissolved compounds or vehicles only. After compound removal and gentle cell washing, complete fresh medium was added in each well and the cells were incubated in the same conditions for a further 24 h. Cells were labelled with 10 mm (final concentration per well) 5-ethynyl-2′-deoxyuridine (EdU) for 30 min. After EdU incubation, media was removed immediately prior to fixation of adherent cells with formaldehyde 3.7% (*v*/*v*) in PBS at room temperature for 15 min. Cells were washed twice with PBS and permeabilized with Triton X-100 (Promega, Madison, MI, USA) for 15 min at room temperature. After two washes with PBS, incorporated EdU was detected with Alexa Fluor 488 by a Click-iT^®^ EdU HCS Assay (C10350, Life Technologies, Grand Island, NY, USA) according to the manufacturer’s recommended procedure. For phospho-histone H3 Ser28 detection, cells were incubated with 3% (*w*/*v*) bovine serum albumin (BSA) overnight at 4 °C, and then incubated for 1 h at room temperature in the dark with the anti-Phospho-Histone H3 primary antibody (Anti-Phospho-Histone H3 pSer28, clone HTA28, SIGMA), 2 µg/mL final concentration in BSA 3%. After three washes with 0.05% Tween^®^ 20, cells were incubated with the secondary antibody (Alexa Fluor^®^ 647 Goat anti-Rat IgG, ThermoFisher Scientific, Waltham, MA, USA), 10 µg/mL final concentration in BSA 3%, for 1 h at room temperature in the dark. Following additional three washes with 0.05% Tween^®^ 20, nuclear staining was performed for 15 min at room temperature in the dark by NuclearMask™ Blue Stain (Life Technologies), 100 µL/well. Finally, following two PBS washes, cells were imaged with an Operetta high-content imaging system (Perkin Elmer) using a 20× long working distance objective. Typically, 10 fields per well were imaged which equated to approximately 6000 cells/well.

### 4.6. Impedentiometric Cell Volume Assay

The reduction of diameter and cell size is a well-known marker of apoptosis in different cell types and tissues [64,65,66]. Cytoplasmic condensation is per se capable to induce apoptosis [67] while cells in active proliferation often show an enhanced diameter size [44]. In our experiments the cells were sized electronically using the Scepter™ 2.0 cell counter (MERK-Millipore, Billerica, MA, USA). Because the Scepter™ cell counter measures volume using the Coulter Principle, it can quantify cells based on size and will discriminate larger cells from smaller debris, unlike vision-based techniques, which rely on object recognition software and cannot reliably detect small cells. In detail, precise cell volumes are drawn into the Scepter™ sensor. As cells flow through the aperture in the sensor, resistance increases. This increase in resistance causes a subsequent increase in voltage. Voltage changes are recorded as spikes with each passing cell and these are proportional to the cell volume. The spikes of the same size are bucketed into a histogram and counted. This histogram gives the quantitative data on cell morphology that can be used to examine the quality and health of the cell culture. The Scepter™ 2.0 cell counter (MERK-Millipore) is compatible with 60 and 40 μm sensors; in our experiments we used the 60 μm sensor for particles between 6 and 36 μm.

### 4.7. Phospo AKT (pSer^473^)/Pan AKT ELISA Assay

The pan AKT and phsphoAKT were measured by an enzyme-linked immunosorbent (ELISA) assay purchased by Sigma-Aldrich (Catalog number RAB0012, SIGMA Chemical Co., Milan, Italy). A pan- AKT antibody has been coated onto a 96-well plate. The samples that were constituted by cell lysates were pipetted into the wells and AKT present in a sample that was bound to the wells by the immobilized antibody. The wells were washed and anti- Phospo AKT (pSer^473^) or pan- AKT were used to detect phosphorylated or pan- AKT, respectively. For statistical results, the assays were run in triplicate. The data from the crude protein preparations were compared with the data obtained from the standard calibration curves performed using the lyophilized positive control sample.

Cell lysates used as samples were prepared according to the instructions reported in the kit datasheet at the end of the 6 h incubation time of SH-SY5Y cells with drugs under investigation, as described in the experimental protocol of the cell volume assay.

### 4.8. Data Analysis and Statistics

Image acquisition and data analysis were performed by using the Harmony^®^ Image Analysis Software with PhenoLOGIC (Perkin Elmer Life Sciences, Boston, MA, USA). Automated analysis was performed to obtain as readout the total number of cells (for information and quality control) and DNA content on a single-cell basis (nuclear mask intensity sum). From the single cell results a histogram of nuclear mask sum was generated (Excel MAC 16.15 software, Microsoft Corp., Redmond, WA, USA) and the intensity corresponding to the center of the G1 peak determined. Based upon this value, a histogram of normalized DNA (relative DNA content) was obtained from vehicle-treated wells on a per-plate basis and applied to all wells to identify G1, S, and G2/M cell populations. Multiparametric analysis of cell-cycle was then performed by using relative DNA content values along with mean marker intensity values of EdU-positive and PHH3-positive cells to specifically identify G1, S, G2, and M cells. In addition, the Harmony Software was used to calculate the nuclear morphological properties (NuclearMask™ Blue Stain), and the nuclear area and fragmentation index of the treated and control cells were then obtained on a single cell level. The fragmentation index was calculated on the basis of the coefficient of variance (CV) of the nuclear stain intensity. Healthy nuclei have a homogeneous intensity, resulting in a low CV value whereas fragmented nuclei have a non-homogeneously distributed intensity resulting in a higher CV value, namely the “fragmentation index”.

The maximal inhibitory effect (E_max_) of the Kv/BK channels targeting compounds on the whole cell K^+^ current was calculated by the following equation:

E_max_ = (*I drug* − *I leak*)/(*I CTRL* − *I leak*) × 100 − 100 (1)

Where *I drug* is the outward current recorded at +30 mV in the presence of the specific drug concentration at which the maximal effect of inhibition of the total K^+^ current was observed; *I leak* is the current recorded at +30 mV in the presence of a saturating concentration of TEA (1 × 10^−3^ M); *I CTRL* is the outward current recorded at +30 mV in the bath solution (without drugs).

The data were collected and analyzed using Excel software (Microsoft Office 2010). The statistical results are presented as mean ± SEM. The number of replicates relative to each experimental data set is reported in the description of the correspondent plot. The Student’s *t*-test and one-way ANOVA were used as appropriate, to evaluate the significance of differences between the means of two or multiple groups, respectively. The one-way ANOVA analysis was completed by the Bonferroni test to assess the experimental groups significantly different from the CTRL. *p* values < 0.05 were considered to indicate a statistical significance.

## 5. Conclusions

In conclusion, the two different blockers of the BK channels IbTX and PAX as well as the BK channel modulator RESV share common actions leading to accumulation in the G2 phase, AKT1p^ser473^ dephosphorylation, and a reduction of cell diameter. This can be related with the block of the surface membrane BK channels. In addition, the action of PAX against intracellular BK channels and/or the interaction with the allosteric side of the pore is associated with G1 accumulation, nuclear area reduction, and the potentiation of its antiproliferative effects with early apoptosis.

## Figures and Tables

**Table 1:** Summary of the effects of the BK channel modulator on cell cycle progression, morphology, and proliferation in SH-SY5Y cells after 6 h of incubation.

Drugs	Mechanism of BK Channel Modulation	Cell Cycle Phase	Nuclear/Cell Morphology	AKT1p^ser473^ Phosporylation	Cell Proliferation
IbTX	Membrane impermeable; BK selective blocker; Open channel blocker; Not reversible action	G2 accumulation; G1 contraction; S not affected	No effects/ Diameter reduction	Dephosphorylation	Moderate reduction
PAX	Membrane permeable; BK selective blocker; Allosteric modulator and closed channel blocker; Not reversible action	G2 accumulation; S contraction; G1 accumulation	Nuclear area shrinking/ Diameter reduction	Marked dephosphorylation	Marked reduction
RESV	Membrane permeable; BK unselective modulator; Not reversible action	G2 accumulation; S contraction	Nuclear area enlargement/ Diameter reduction	Dephosphorylation	Moderate reduction
TEA	Kv/BK unselective blocker with reversible action	No effects	No Effects/No Effects	No effects	No effects
